# Socioeconomic status can affect pregnancy outcomes and complications, even with a universal healthcare system

**DOI:** 10.1186/s12939-017-0715-7

**Published:** 2018-01-05

**Authors:** Min Kyoung Kim, Seung Mi Lee, Sung-Hee Bae, Hyun Joo Kim, Nam Gu Lim, Seok-Jun Yoon, Jin Yong Lee, Min-Woo Jo

**Affiliations:** 1 Department of Obstetrics and Gynecology, Seoul National University College of Medicine, Seoul, Korea; 2 Public Health Medical Service, Boramae Medical Center, Seoul National University College of Medicine, Seoul, Korea; 3 Department of Nursing Science, Shinsung University, Dangjin, Korea; 4 Department of Medical Administration and Information, Daejeon Health Institute of Technology, Daejeon, Korea; 5 Department of Preventive Medicine, College of Medicine, Korea University, Seoul, Korea; 6 Institute of Health Policy and Management, Medical Research Center, Seoul National University, Seoul, Korea; 7 Department of Preventive Medicine, University of Ulsan College of Medicine, Seoul, Korea

**Keywords:** Prenatal care, Preterm delivery, National Health Insurance, Socioeconomic status, Health equity

## Abstract

**Background:**

Low socioeconomic status can increase the risk of adverse pregnancy outcomes, but it remains unclear whether this negative association is attributed to inadequate prenatal care. Korea has been adopting a universal healthcare system. All Korean citizens must be enrolled National Health Insurance (NHI) or be recipient of Medical Aid (MA). In addition, Korean government launched a financial support system for antenatal care for all pregnant women in 2008. Therefore, in theory, there is no financial barrier to receive prenatal cares regardless of someone’s social class. However, it is still unclear whether adverse pregnancy outcomes observed in low-income women are attributable to low SES or to economic barriers specific to the utilization of medical services. The purpose of this study was to investigate whether socioeconomic status affects pregnancy outcomes after the introduction of this support system, which allows all pregnant women to receive adequate prenatal care regardless of socioeconomic status.

**Methods:**

Using the National Health Insurance database in Korea, we selected women who gave birth between January 1, 2010 and December 31, 2010. As a proxy indicator reflecting socioeconomic status, we classified subjects as MA recipient (“low” SES) or a NHI beneficiary (“middle/high” SES).

**Results:**

In the MA group, 29.4% women received inadequate prenatal care, compared to 11.4% in the NHI group. Mothers in the MA group were more likely to have an abortion (30.1%), rather than deliver a baby, than those in the NHI group (20.7%, *P* < 0.001). Mothers in the MA group were also more likely to undergo a Caesarean delivery (45.8%; NHI group: 39.6%, *P* < 0.001), and have preeclampsia (1.5%; NHI group: 0.6%, *P* < 0.001), obstetric hemorrhage (4.7%; NHI group: 3.9%, *P* = 0.017), and a preterm delivery (2.1%; NHI group: 1.4%, *P* < 0.001) than those in the NHI group.

**Conclusions:**

Women in the MA group tended to show higher rates of abortion, Caesarean delivery, preeclampsia, preterm delivery, and obstetrical hemorrhage than those in the NHI group Therefore, health authorities should consider investigating what kind of barriers exist or what factors may affect these inequitable outcomes.

## Background

Socioeconomic status (SES) is one of the most important factors associated with medical outcomes. When SES is low, medical care is inadequate and this has been attributed to adverse outcomes [1, 2]. In pregnant women, low SES can increase the risk of adverse pregnancy outcomes. Previous studies have revealed that low SES is associated with pregnancy complications such as abortion, preterm delivery, preeclampsia, eclampsia, and gestational diabetes [3–6]. Inadequate prenatal care is associated with poor obstetric outcomes, including preterm delivery, preeclampsia, and stillbirth [1, 4–7], and women with low SES are less likely to receive prenatal care [1, 2, 8]. In fact, the risk of preterm delivery, preeclampsia, and gestational diabetes increases with both inadequate prenatal care and low SES [3–5].

In Korea, the healthcare security system is divided into two sections according to income: the Medical Aid (MA) system for low-income individuals and the National Health Insurance (NHI) for middle/high-income individuals [9]. According to statistics from the Korean government, the MA system covered 2.9% of people in 2014, providing access to healthcare at “a minimum cost” to low-income individuals.

In addition to MA and NHI, the Korean government introduced additional financial support for antenatal care for all pregnant women in 2008. All pregnant women were provided with a credit card, named the GOUNMOM card (GOUNMOM translates to “good mother” in Korean), to subsidize medical expenses for pregnancy and childbirth. The GOUNMOM card is akin to a welfare voucher, whereby pregnant women can spend approximately $500 up to 60 days after childbirth, at a maximum of $60 per day [10]. This economic support diminishes the financial burden for women, especially MA recipients. In theory, this Korean insurance system allows for all pregnant women to receive adequate prenatal care without economic barriers.

However, it is still unclear whether adverse pregnancy outcomes observed in low-income women are attributable to low SES or to economic barriers specific to the utilization of medical services. Until now, SES had not been investigated as an obstetrical risk factor independent of inadequate medical care. To clarify this association, we investigated whether SES might affect pregnancy outcomes after the introduction of an additional financial support program: the GOUNMOM card. This allowed us to compare pregnancy outcomes according to SES in the absence of financial barriers.

## Methods

### Data sources and study population

We used the database from the National Health Insurance Service (NHIS), which is the sole healthcare insurer in Korea. The study subjects were 461,580 women (aged 20 years and older) who gave birth between January 1, 2010 and December 31, 2010. We used the type of health insurance, specifically NHI or MA, as a proxy indicator reflecting SES. Among the 461,580 women, 99.1% (*n* = 457,336) were NHI beneficiaries and 0.9% (*n* = 4244) were MA recipients.

### Pregnancy-related indicators

Our main hypothesis was that there is no difference in pregnancy-related indicators (PRIs) between NHI beneficiaries and MA recipients. The PRIs included in this study were prenatal care utilization, obstetric outcomes, and the occurrence of obstetric complications. With the exception of prenatal care utilization, all other PRIs were defined based on the International Classification of Diseases, 10th Revision (ICD-10) (Table 1).

**Table 1:** The definition of abortion, delivery, and obstetric complications

Variables	ICD-10 code
(1) Abortion	O00-O08 (O00-O089)
(2) Delivery	
2-1) Cesarean section	O82 (O820-O829), O842
2-2) Vaginal delivery	O80 (O800-O809), O81 (O810-O815), O83 (O830-O839), O840, O841
(3) Obstetric complications	
3-1) Preeclampsia	O14 (O140-O149)
3-2) Eclampsia	O15 (O150-O159)
3-3) Gestational hypertension	O13
3-4) Gestational diabetes mellitus	O244
3-5) Placenta previa	O44 (O440-O441)
3-6) Abruptio placentae	O45 (O450-O459)
3-7) Obstructed labor	O64-O66 (O640-O669)
3-8) Preterm delivery	O601
3-9) Acute pyelonephritis	O23 (O230-O239), N10, N12, N159
3-10) Perineal laceration	O702, O703
3-11) Obstetric hemorrhage	O67 (O670-O679), O72 (O720-O723)

To evaluate the adequacy of prenatal care utilization, we used a modified version of the Kessner index [11], which is based on the total number of prenatal care visits during pregnancy (“adequate”, 9 occasions or more; “intermediate”, 5–8 occasions; and “inadequate”, fewer than 5 times). To compare the NHI and MA groups, their basic health status was adjusted; that is, if there were differences in PRIs, we considered the effect of their basic health status. Therefore, we used the Charlson comorbidity index to adjust for the effect of basic health status [12–14].

### Statistical analysis

Frequency analyses were performed to describe PRIs. Pearson’s chi-square test and the student’s *t*-test were used to examine the differences between the NHI and MA groups. To define the factors associated with abortion and maternal complications, multivariate analyses were performed using logistic regression. All analyses were completed using SPSS, version 19.0 (IBM, Chicago, IL, USA). All statistical tests were two-sided and a *P* value <0.05 was considered statistically significant.

## Results

### General characteristics of study subjects

Compared to women in the NHI group, women in the MA group were slightly older (32.7 vs. 31.6 years, respectively) (*P* < 0.001) and more likely to live in rural areas (12.7% vs. 8.1%, respectively) (*P* < 0.001). According to the Charlson comorbidity index, there was no significant difference in health status between the groups (*P* = 0.112) (Table 2).

**Table 2:** General characteristics of subjects

Variables	No. (%) of women			
	NHI	MA	Total	*P value*
Total	457,336 (99.1)	4244 (0.9)	461,580 (100)	
Age^*^				
Mean ± SD	31.6 ± 4.29	32.7 ± 6.57	31.6 ± 4.31	<0.001
20-34	350,977(76.7)	2399(56.5)	353,376(76.6)	<0.001
≥ 35	106,359(23.3)	1845(43.5)	108,204(23.4)	
Area^*^				<0.001
Metropolitan	192,006 (42.0)	1687 (39.8)	193,693 (42.0)	
City	228,394(49.9)	2020(47.6)	230,414 (49.9)	
Rural	36,936 (8.1)	537 (12.7)	37,473 (8.1)	
Level of Income^*, a^				<0.001
High (upper 25%)	115,589 (25.3)	0 (0.0)	115,589 (25.0)	
Medium (25-75%)	229,966 (50.3)	0 (0.0)	229,966 (49.8)	
Low (lower 25%)	111,781 (24.4)	4244(100)	116,025 (25.1)	
Charlson comorbidity index				0.112
0	451,963(98.8)	4183(98.6)	456,146(98.8)	
≥ 1	5373(1.2)	61(1.4)	5434(1.2)	

*No* number, *NHI* National Health Insurance, *MA* Medical Aid, *SD* Standard Deviation

^a^Level of income was categorized as high level (upper 25% of premium), intermediate level (middle 50% of premium), and low level (lower 25% of premium) based on their national health insurance premium

^*^P < 0.05 calculated by chi-square test or *t*-test

### Prenatal care utilization

Regarding the frequency of prenatal care, the mean number of visits to the doctor in women in the MA group was 7.3, which is 2 visits less than in women in the NHI group, who visited their doctors a mean of 9.4 times (*P* < 0.001). The proportion of mothers who received adequate prenatal care was 37.5% in the MA group, which was less than in the NHI group (54.8%) (Table 3). Indeed, about 7.2% of mothers in the MA group had never received prenatal care at any point during pregnancy.

**Table 3:** Prenatal care utilization and obstetric outcomes

Variables	No. (%) of women			
	NHI (*n* = 457,336)	MA (*n* = 4244)	Total	*P value*
Prenatal care utilization				
Frequency of prenatal care utilization	9.4 ± 4.44	7.3 ± 4.69	9.4 ± 4.44	<0.001
Adequacy of prenatal care utilization				<0.001
Adequate (≥ 9)	198,623 (54.8)	1113 (37.5)	199,736 (54.6)	
Intermediate (5–8)	122,609 (33.8)	982 (33.1)	123,591 (33.8)	
Inadequate (≤ 4)	41,379 (11.4)	873 (29.4)	42,252 (11.6)	
Obstetric outcome				*P value*
Termination of pregnancy^*^				<0.001
Abortion	94,725 (20.7)	1276 (30.1)	96,001 (20.8)	
Delivery	362,611 (79.3)	2968 (69.9)	365,579 (79.2)	
Result of childbirth^*^				0.025
Live birth	361,967 (99.8)	2957 (99.6)	364,924 (99.8)	
Stillbirth	644 (0.2)	11 (0.4)	655 (0.2)	
Subtotal	362,611 (99.2)	2968 (0.8)	365,579 (100)	
Type of delivery^*^				<0.001
Cesarean section	143,558 (39.6)	1360 (45.8)	144,918 (39.6)	
Vaginal delivery	219,053 (60.4)	1608 (54.2)	220,661 (60.4)	
Subtotal	362,611 (99.2)	2968 (0.8)	365,579 (100)	

*No* number, *NHI* National Health Insurance, *MA* Medical Aid, *SD* Standard Deviation

^*^P < 0.05 calculated by chi-square test or *t*-test

### Obstetric outcomes and occurrence of obstetric complications

Mothers in the MA group showed higher rates of abortion (30.1% vs. 20.7%, *P* < 0.001) and stillbirth (0.4% vs. 0.2%, *P* = 0.025) than mothers in the NHI group. The possibility of Caesarean delivery was also higher (45.8% vs. 39.6%, *P* < 0.001). Insurance through MA remained a significant risk factor for abortion and Caesarean delivery, even after adjustment for maternal age, region, and the Charlson comorbidity index (Table 4).

**Table 4:** Factors associated with abortion and Cesarean section

Variables	Abortion		Cesarean delivery	
	aOR (95% CI)	*P* value	aOR (95% CI)	*P* value
Age (year)				
20-34	1.00		1.00	
≥ 35	2.22 (2.19-2.25)	<0.001	1.85 (1.82-1.88)	<0.001
Region				
Metropolitan	1.00		1.00	
City	0.96 (0.95-0.98)	<0.001	1.06 (1.04-1.07)	<0.001
Rural	1.02 (0.99-1.05)	0.164	1.13 (1.10-1.16)	<0.001
Type of insurance				
National Health Insurance	1.00		1.00	
Medical aid	1.40 (1.31-1.50)	<0.001	1.17 (1.08-1.25)	<0.001
Charlson comorbidity index				
0	1.00		1.00	
≥ 1	0.49 (0.45-0.53)	<0.001	1.27 (1.20-1.35)	<0.001

*aOR* adjusted odds ratio, *CI* confidence interval

We conducted univariate analysis to investigate whether the incidence of obstetric complications differed between the NHI group and the MA group. The risks of preeclampsia (1.5% vs. 0.6%, *P* < 0.001), eclampsia (0.1% vs. 0.0%, *P* = 0.005), gestational diabetes mellitus (1.0% vs. 0.6%, *P* = 0.016), preterm delivery (2.1% vs. 1.3%, *P* < 0.001), and obstetric hemorrhage (4.7% vs. 3.9%, *P* = 0.017) were higher in mothers in the MA group. In contrast, women in the NHI group (1.6%) had a higher risk of perineal laceration than those in the MA group (0.9%) (*P* = 0.005) (Table 5).

**Table 5:** Obstetric complications

Obstetric complications	No. (%) of women			
	NHI (*n* = 457,336)	MA (*n* = 4244)	Total	*P value*
Preeclampsia^*^	2247 (0.6)	44 (1.5)	2291 (0.6)	<0.001
Eclampsia^*^	82 (0.0)	4 (0.1)	86 (0.0)	0.005
Gestational hypertension	729 (0.2)	7 (0.2)	736 (0.2)	0.677
Gestational diabetes mellitus^*^	2338 (0.6)	30 (1.0)	2368 (0.6)	0.016
Placenta previa	2370 (0.7)	22 (0.7)	2392 (0.7)	0.566
Abruptio placentae	822 (0.2)	4 (0.1)	826 (0.2)	0.340
Obstructed labor	14,469 (4.0)	130 (4.4)	14,599 (4.0)	0.300
Preterm delivery^*^	4728 (1.3)	62 (2.1)	4790 (1.3)	<0.001
Acute pyelonephritis	537 (0.1)	4 (0.1)	541 (0.1)	1.000
Perineal laceration^*^	5668 (1.6)	27 (0.9)	5695 (1.6)	0.005
Obstetric hemorrhage^*^	13,967 (3.9)	140 (4.7)	14,107 (3.9)	0.017

*No* number, *NHI* National Health Insurance, *MA* Medical Aid

^*^P < 0.05 calculated by chi-square test

We then performed multivariate analyses using logistic regression to investigate which factors affected each maternal complication. Table 6 shows that even after adjusting for other factors, insurance through MA was a significant risk factor for preterm delivery (odds ratio [OR]: 1.35, *P* = 0.022), preeclampsia (OR: 1.98, *P* < 0.001), gestational diabetes mellitus (OR: 1.43, *P* = 0.055), and obstetric hemorrhage (OR: 1.25, *P* = 0.010), but not for perineal laceration (OR: 0.63, *P* = 0.019) (Table 6).

**Table 6:** Factors associated with obstetric complications

Variables	Preterm delivery		Preeclampsia		Gestational diabetes mellitus		Perineal laceration		Obstetric hemorrhage	
	aOR (95% CI)	*P* value	aOR (95% CI)	*P* value	aOR (95% CI)	*P* value	aOR (95% CI)	*P* value	aOR (95% CI)	*P* value
Age (year)										
20-34	1.00		1.00		1.00		1.00		1.00	
≥ 35	1.16(1.08-1.24)	<0.001	1.49(1.36-1.64)	<0.001	1.99(1.82-2.17)	<0.001	0.71(0.65-0.77)	<0.001	1.06(1.02-1.11)	0.005
Area										
Metropolitan	1.00		1.00		1.00		1.00		1.00	
City	0.95(0.90-1.01)	0.123	0.97(0.89-1.06)	0.488	0.79(0.73-0.86)	<0.001	1.33(1.26-1.41)	<0.001	0.80(0.77-0.83)	<0.001
Rural	1.08(0.97-1.20)	0.157	1.29(1.11-1.48)	0.001	0.86(0.73-1.01)	0.058	1.29(1.17-1.43)	<0.001	1.44(1.37-1.53)	<0.001
Type of insurance										
National Health Insurance	1.00		1.00		1.00		1.00		1.00	
Medical aid	1.35(1.04-1.74)	0.022	1.98(1.46-2.67)	<0.001	1.43(0.99-2.06)	0.055	0.66(0.45-0.97)	0.033	1.28(1.08-1.52)	0.005
Charlson comorbidity index										
0	1.00		1.00		1.00		1.00		1.00	
≥ 1	1.41(1.13-1.76)	0.002	1.99(1.53-2.59)	<0.001	9.22(8.08-10.52)	<0.001	0.59(0.43-0.81)	0.001	1.19(1.04-1.37)	0.013
Adequacy of prenatal care										
Adequate (≥ 9)	1.00		1.00		1.00		1.00		1.00	
Intermediate (5–8)	1.34(1.26-1.43)	<0.001	1.03(0.93-1.13)	0.593	0.88(0.80-0.96)	0.004	0.69(0.65-0.74)	<0.001	0.73(0.70-0.76)	<0.001
Inadequate (≤ 4)	2.16(1.99-2.33)	<0.001	1.73(1.55-1.94)	<0.001	0.83(0.72-0.95)	0.009	1.01(0.92-1.09)	0.896	0.86(0.81-0.91)	<0.001
Cesarean section										
Yes							1.00		1.00	
No (vaginal delivery)							1871.61(467.96-7485.59)	<0.001	2.02(1.94-2.10)	<0.001

*aOR* adjusted odds ratio, *CI* confidence interval

## Discussion

The principal findings of the current study are as follows: 1) women in the MA group received prenatal care less frequently than those in the NHI group; 2) in terms of obstetric outcomes, mothers in the MA group were more likely to have an abortion or a Caesarean delivery than those in the NHI group; and 3) the risk of obstetric complications, such as preeclampsia, gestational diabetes, preterm delivery, and obstetric hemorrhage, was significantly increased in women in the MA group, even after adjustment for adequacy of prenatal care.

The findings that individuals with lower SES tend to receive prenatal care less frequently and are at higher risk for obstetric complications are consistent with the findings of previous studies [1, 2]. Individuals with low SES tend to be disadvantaged in terms of medical service utilization [1, 2, 15]. Pregnant women with low SES have been shown to have adverse obstetric outcomes associated with inadequate prenatal visits [1, 4, 6, 15]. The results of the current study that obstetric complications were poor in women in the MA group is consistent with the results of previous studies demonstrating that low SES is a risk factor for poor obstetric outcomes [1–6]. Our study evaluated the correlation of SES and obstetric outcomes in Korea with its unique medical insurance system. Recently, the Korean government launched a financial support program, named the GOUNMOM card. With this card, the financial gap between MA recipients and NHI beneficiaries was eliminated, providing individuals with low SES an equal opportunity to medical care under the MA system [10, 16–18]. In this way, every pregnant woman could receive adequate prenatal care regardless of SES. The purpose of this study was to examine SES as a risk factor for poor obstetric outcomes, independent of medical expense burden.

We found that obstetric outcomes, including abortion, stillbirth, and Caesarean delivery rates, were higher among women in the MA group than among those in the NHI group, even after the introduction of the GOUNMOM card. This is different from a previous study in Kenya, wherein the barrier to medical access is much more substantial than in Korea [16, 17]. In addition, the risk of obstetric complications remained elevated in the MA group after adjustment for adequacy of prenatal care and the Charlson comorbidity index (Table 6). These results suggest that other factors may be associated with adverse pregnancy outcomes in women with low SES.

First, occupational factors, such as long working hours and physical exertion, likely affect obstetric outcomes. Prolonged working hours or occupational fatigue are risk factors for preterm birth and preeclampsia [5, 19]. Increased working hours per day could be a barrier to adequate prenatal visits, which are directly associated with severe complications: notably, preeclampsia, preterm labor, and gestational diabetes [1, 2]. Second, other economic factors like costs of transportation to the hospital and the opportunity cost of receiving medical care may be a sufficient burden that restricts prenatal care in pregnant women with low SES. Third, low educational level is related to the probability of seeking antenatal care inappropriately. Educated women tend to receive antenatal check-ups more frequently than less educated women [20]. Lastly, the adequate prenatal visit rate was lower in the MA group (37.5%) compared with the NHI group (54.8%), despite MA supporting medical expenses.

Therefore, to improve obstetric outcomes in low-income women, fundamental support to receive more prenatal care or to modify lifestyle risk factors, such as long working hours, may be needed in addition to lowering the burden of medical expenses. This finding suggests that the national health policy should not solely concentrate on the enhancement of adequate prenatal care. Instead, there is a need for social interventions aimed at more in-depth and distal determinants of health to improve pregnancy outcomes in pregnant women with low SES.

In the current study, the frequency of preeclampsia, preterm birth, and gestational diabetes was slightly lower than that previously reported. In Korea, the reported rates of preterm delivery, preeclampsia, and gestational diabetes are 5.9%, 2.2%, and 2.5%, respectively [21–23]. These differences may be related to the definition of each complication, or to the differences in study populations or study periods.

This study has some limitations and we need to perform further research on aspects that could not be investigated in the current study. First, this study could not determine whether the new policy implemented in 2008 had a positive impact on beneficiaries (i.e., whether the policy affected prenatal care uptake among those with low SES). This is a very critical issue in terms of policy implementation and should be investigated in the near future. Second, there is a possible critique that comparing MA (people with low SES) and NHI (everyone else) recipients is too crude of a measurement method. For future research, we will consider more detailed economic class segmentations, such as by health insurance premiums. Lastly, the analyzed number of MA recipients was lower compared to the number of NIH beneficiaries. Thus, further research with a large number of MA recipients is needed for evaluation of the quality of antenatal care.

## Conclusions

We determined that SES can affect pregnancy outcomes even under a universal healthcare system. MA recipients tend to show higher rates of abortion, Caesarean delivery, preeclampsia, preterm delivery, and obstetrical hemorrhage than NHI beneficiaries. Therefore, health authorities should consider investigating the kinds of barriers that exist and the factors affecting these inequitable outcomes.

## Abbreviations

MA: Medical aid
NHI: National health insurance
NHIS: National health insurance service
SES: Socioeconomic status
